# Multidisciplinary Development and Initial Validation of a Clinical Knowledge Base on Chronic Respiratory Diseases for mHealth Decision Support Systems

**DOI:** 10.2196/45364

**Published:** 2023-12-13

**Authors:** Ana Margarida Pereira, Cristina Jácome, Tiago Jacinto, Rita Amaral, Mariana Pereira, Ana Sá-Sousa, Mariana Couto, Pedro Vieira-Marques, Diogo Martinho, Ana Vieira, Ana Almeida, Constantino Martins, Goreti Marreiros, Alberto Freitas, Rute Almeida, João A Fonseca

**Affiliations:** 1 Department of Community Medicine, Information and Health Decision Sciences (MEDCIDS) Faculty of Medicine University of Porto Porto Portugal; 2 Allergy Unit Instituto and Hospital CUF-Porto Porto Portugal; 3 PaCeIT – Patient Centered Innovation and Technologies, Center for Health Technology and Services Research Faculty of Medicine University of Porto Porto Portugal; 4 CINTESIS@RISE, Department of Community Medicine, Information and Health Decision Sciences (MEDCIDS) Faculty of Medicine University of Porto Porto Portugal; 5 Department of Cardiovascular and Respiratory Sciences Porto Health School Polytechnic Institute of Porto Porto Portugal; 6 Department of Women’s and Children’s Health Pediatric Research Uppsala University Uppsala Sweden; 7 MEDIDA – Medicina, Educação, Investigação, Desenvolvimento e Avaliação Porto Portugal; 8 Allergy Center CUF Descobertas Hospital Lisboa Portugal; 9 Research Group on Intelligent Engineering and Computing for Advanced Innovation and Development Institute of Engineering Polytechnic of Porto Porto Portugal

**Keywords:** knowledge base, recommendations, personalization, clinical decision support system, chronic obstructive respiratory diseases, mobile phone

## Abstract

Most mobile health (mHealth) decision support systems currently available for chronic obstructive respiratory diseases (CORDs) are not supported by clinical evidence or lack clinical validation. The development of the knowledge base that will feed the clinical decision support system is a crucial step that involves the collection and systematization of clinical knowledge from relevant scientific sources and its representation in a human-understandable and computer-interpretable way. This work describes the development and initial validation of a clinical knowledge base that can be integrated into mHealth decision support systems developed for patients with CORDs. A multidisciplinary team of health care professionals with clinical experience in respiratory diseases, together with data science and IT professionals, defined a new framework that can be used in other evidence-based systems. The knowledge base development began with a thorough review of the relevant scientific sources (eg, disease guidelines) to identify the recommendations to be implemented in the decision support system based on a consensus process. Recommendations were selected according to predefined inclusion criteria: (1) applicable to individuals with CORDs or to prevent CORDs, (2) directed toward patient self-management, (3) targeting adults, and (4) within the scope of the knowledge domains and subdomains defined. Then, the selected recommendations were prioritized according to (1) a harmonized level of evidence (reconciled from different sources); (2) the scope of the source document (international was preferred); (3) the entity that issued the source document; (4) the operability of the recommendation; and (5) health care professionals’ perceptions of the relevance, potential impact, and reach of the recommendation. A total of 358 recommendations were selected. Next, the variables required to trigger those recommendations were defined (n=116) and operationalized into logical rules using Boolean logical operators (n=405). Finally, the knowledge base was implemented in an intelligent individualized coaching component and pretested with an asthma use case. Initial validation of the knowledge base was conducted internally using data from a population-based observational study of individuals with or without asthma or rhinitis. External validation of the appropriateness of the recommendations with the highest priority level was conducted independently by 4 physicians. In addition, a strategy for knowledge base updates, including an easy-to-use rules editor, was defined. Using this process, based on consensus and iterative improvement, we developed and conducted preliminary validation of a clinical knowledge base for CORDs that translates disease guidelines into personalized patient recommendations. The knowledge base can be used as part of mHealth decision support systems. This process could be replicated in other clinical areas.

## Introduction

More than 1 billion individuals worldwide have chronic respiratory diseases [1]. Chronic obstructive respiratory diseases (CORDs) are high-burden chronic diseases throughout the life cycle—asthma usually starting at an early age and chronic obstructive pulmonary disease (COPD) starting from middle age onward, having major adverse effects on the life and disability of patients. Patients with CORDs are continuously at risk of health deterioration, requiring regular medical visits. Furthermore, in between visits, patients manage their health in their environment without immediate support from a health care professional and mostly based on traditional self-management materials (books, leaflets, videos, and web-based technology).

Mobile apps are promising for improving self-management behaviors in patients with CORDs as they can be easily integrated into the patients’ everyday lives [2]. Worldwide, the global smartphone penetration rate is estimated to be 68%, with 6.3 billion smartphone users [3]. Patients with CORDs are as likely to own a smartphone as members of the general population [4,5]. Ownership levels are approximately 40% in older patients [6] and 80% in younger ones [4], and these rates are likely to increase in the future. Smartphones have the advantages of being personal, portable, connected, (increasingly) low cost, and computationally powerful. It is hypothesized that apps may be ubiquitous solutions capable of improving and maintaining self-management behaviors in the long run and having an impact on a large number of patients.

On the basis of this assumption, apps for CORD self-management are currently available in web stores [7-9] and have been shown to have a positive effect on symptom control, medication adherence, and self-efficacy [10,11]. However, asthma apps only capture <1% of the target market [12]. Indeed, in a study conducted by our team, only 3% of the participants had ever used an app directly related to their asthma [4]. This result may be explained by the lack of patient and health professional knowledge of existing apps and their benefits for asthma management. In addition, it may also be related to the fact that most asthma apps are exclusively tracker apps, do not enable automated data input or personalized feedback, and do not provide behavior change support or coaching solutions [2,12]. We did not find data on COPD app market penetration, but there is no reason to expect it to be better than what has been reported for asthma. A review of COPD apps found that features to enhance behavior changes, such as social networking tools, personalized education, feedback, coaching, and psychological motivation, were also missing in most of the analyzed apps [9]. Furthermore, most asthma and COPD mobile health (mHealth) solutions currently available are not supported by clinical evidence or lack clinical validation [13].

To our knowledge, a scientifically grounded, structured knowledge base to be used in mHealth systems supporting CORD self-management is lacking. Under the scope of the AIRDOC (Aplicação móvel Inteligente para suporte individualizado e monitorização da função e sons Respiratórios de Doentes Obstrutivos Crónicos) project [14], we aimed to develop and validate an attractive integrated mHealth system for the self-management of CORDs with a scientifically grounded coaching component based on a clinical decision support system (CDSS). The CDSS will combine patient-processed data with a clinical knowledge base to provide individualized support to adult patients with CORDs. The knowledge base to be used in the AIRDOC ecosystem was developed by a multidisciplinary team using a structured process that is intended to make it more consistent and potentially useful to other mHealth systems. This paper aims to describe the process of development and initial validation of a CDSS knowledge base supporting CORD self-management.

Specifically, we aimed to (1) identify and retrieve management-related recommendations from the review of relevant international and national guidelines on CORDs, (2) operationalize the knowledge base by identifying the required information and logical rules that trigger each personalized recommendation, (3) validate the knowledge base using real-world data from clinical studies and an external physician group, and (4) define the process to update the knowledge base to accommodate future changes in CORD guidelines.

The next section describes the framework for the development of the CDSS knowledge base and will be followed by a detailed description of the steps involved in this process.

## Definition of a Framework for the Development of the CDSS Knowledge Base

CDSSs can be defined as “software that is designed to be a direct aid to clinical decision-making in which the characteristics of an individual patient are matched to a computerized clinical knowledge base, and patient-specific assessments or recommendations are then presented to the clinician and/or the patient for a decision” [15]. A CDSS provides knowledge and person-specific information adequately filtered and presented at appropriate times to enhance health care delivery [16].

**Figure 1:**
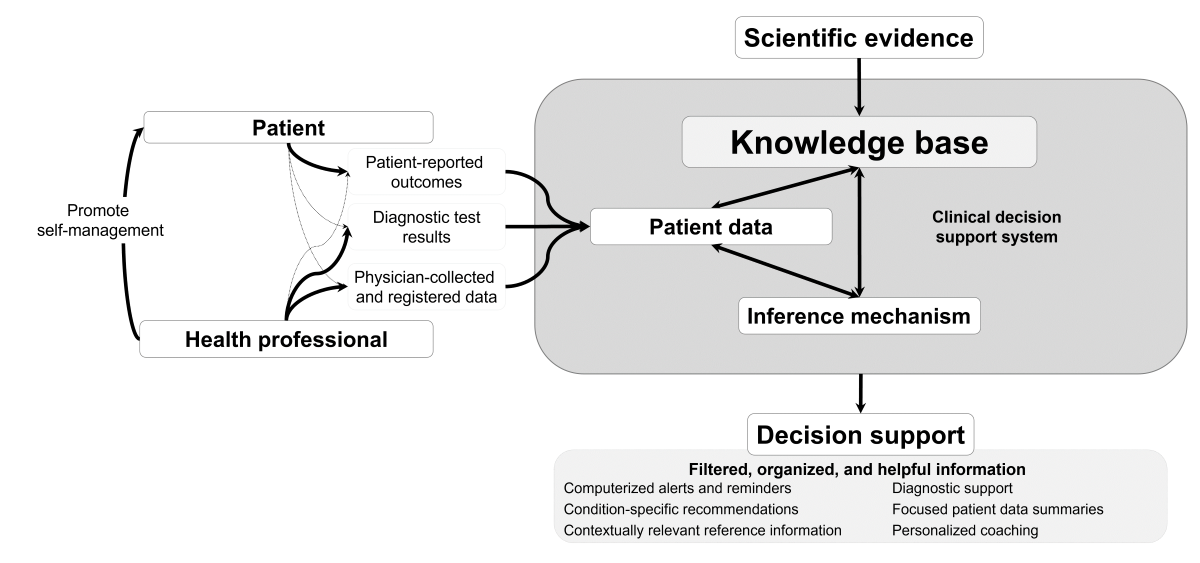
Clinical decision support systems use a knowledge base, an inference mechanism, and patient data from one or several sources to provide different forms of decision support. Adapted from Pereira et al [22].

A CDSS has three main architectural components [17-19]: (1) a knowledge base that sums up the available scientific evidence on the area of interest, (2) patient data (eg, medical history, diseases, symptoms, and treatment plans), and (3) an inference mechanism (eg, a prediction rule, an algorithm, Bayesian networks, or machine learning) to process the patient data according to the clinical knowledge base. This interaction leads to the provision of decision support, which can have several forms (eg, alerts and reminders or diagnostic support) and targets (eg, patients and health care professionals; Figure 1).

Building the knowledge base is a crucial step that can determine the success of the entire CDSS [17]. According to Purcell [21], a CDSS is as effective as its underlying knowledge base. The construction of the knowledge base should account for the requirements of all other CDSS components and requires special attention from the earliest stage of CDSS design until its final stages of implementation and validation [17]. This involves the collection and systematization of clinical knowledge from relevant scientific sources and its representation in a human-understandable and computer-interpretable way [17,22] so that it can be used by the inference mechanism.

**Figure 2:**
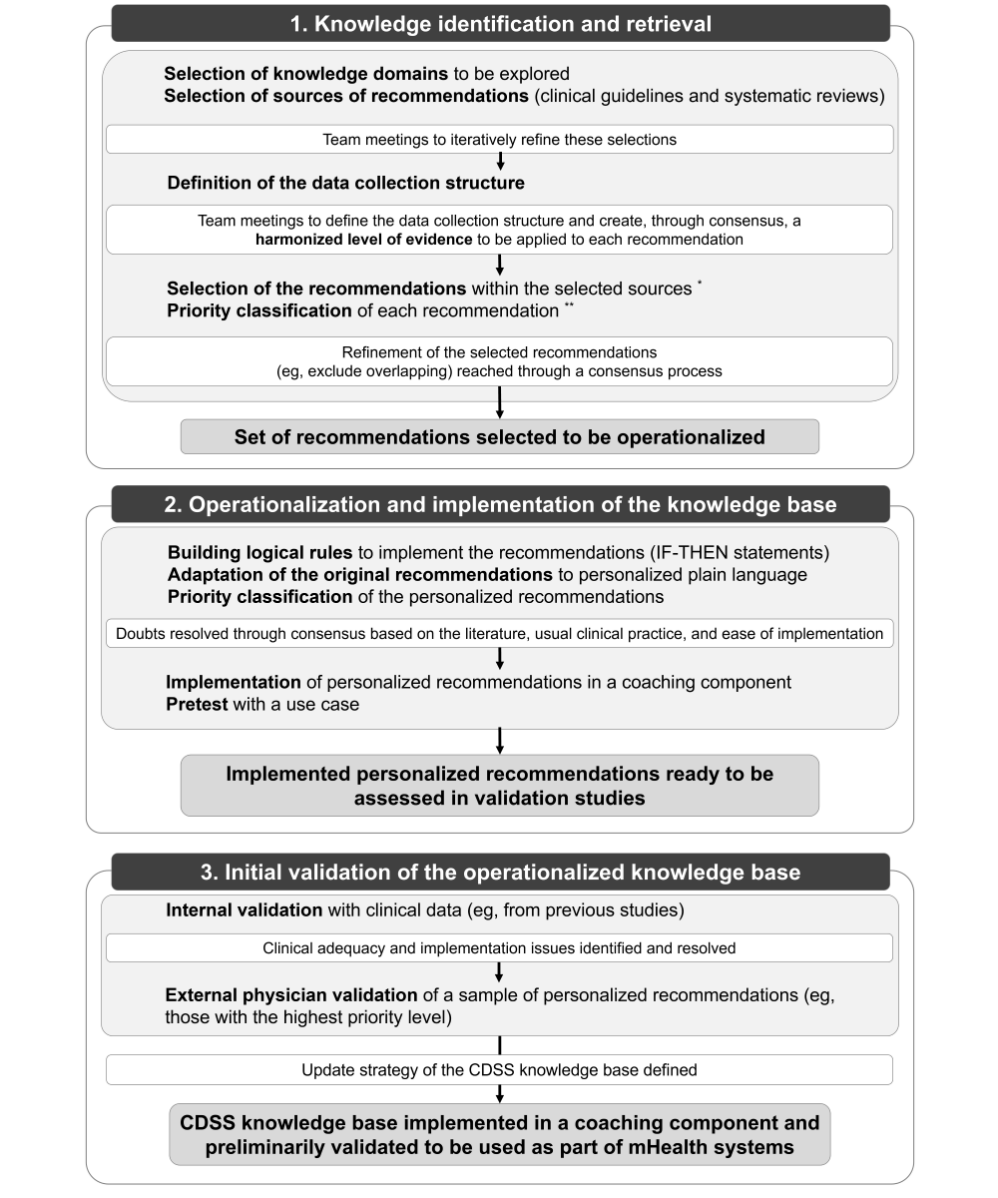
Steps in the development and initial assessment of the knowledge base. This knowledge base was developed in three major steps: (1) knowledge identification and retrieval, (2) operationalization and implementation, and (3) initial validation of the operationalized knowledge base. A complete description of each step is presented in the text. *Inclusion criteria: (1) applicable to individuals with chronic obstructive respiratory diseases (CORDs) or to prevent CORDs, (2) directed toward patient self-management, (3) targeting adults, and (4) within the scope of the selected domains and subdomains. **Prioritization criteria: (1) harmonized level of evidence; (2) scope of the source document (international recommendations were preferred); (3) entity that issued the source document; (4) operability of the recommendation (eg, type of data that would be needed to implement it in a personalized way); and (5) health care professionals’ perceptions of the relevance, potential impact, and reach of the recommendation. CDSS: clinical decision support system; mHealth: mobile health.

In the next sections, we will describe the process of gathering scientific knowledge for supporting the self-management of CORDs and its operationalization and implementation through decision support rules designed to feed the CDSS inference mechanism and help define the needs regarding input patient data. The steps followed in the development of this knowledge base are illustrated in Figure 2. The framework used for the development of the CDSS knowledge base was defined by a team of health care, data sciences, and IT professionals in a series of meetings held in September 2018. The development of the knowledge base was conducted by this multidisciplinary team between September 2018 and March 2019 and involved 2 to 3 monthly face-to-face or videoconference meetings. The health care professionals had different backgrounds (allergists, clinical physiologists, physiotherapists, and pharmacists) and had clinical and academic experience.

## Knowledge Identification and Retrieval

The process of knowledge acquisition is the first and most important step in building the knowledge base [17]. Before identifying and retrieving the recommendations, the relevant knowledge domains and the most important sources were selected, and the data collection structure was defined.

### Selection of Domains and Sources of Recommendations

A total of 6 in-person meetings were held (between September 2018 and October 2018) until a comprehensive list of domains, subdomains, and sources of recommendations was approved through consensus. These lists were iteratively refined during the process of identification, retrieval, and prioritization of the recommendations.

The domains relevant to the management of COPD and asthma were initially suggested by health care professionals in the knowledge base development team. Some of the initial domains and subdomains were then reorganized (new subdomains were created and some were merged or excluded) based on the detailed analysis of the individually selected recommendations and their global relevance and priority (Table 1).

The main sources of recommendations were the Global Initiative for Chronic Obstructive Lung Disease (GOLD) [23] and the Global Initiative for Asthma (GINA) [24] reports as these represent the most important and renowned international guidelines for the diagnosis and management of COPD and asthma, respectively. Other national and international guidelines were used as complementary sources of recommendations, mostly because of their relevance in different domains of the management of CORDs and associated multimorbidity, such as physical activity and exercise, allergic rhinitis, adherence to treatment, and airway clearance (Table 2). These additional guidelines were selected by the team based on their perceived relevance and international impact (eg, publication in international indexed journals, strength of the development process, and frequency of use as a reference in articles published in international indexed journals) and the potential to have additional recommendations that could be applied to a broad population. When necessary, other data sources (eg, systematic reviews and meta-analyses) were used to complement the information, thereby strengthening the recommendations on some aspects that were not fully covered by the guidelines.

**Table 1:** Relevant domains and subdomains, including the initial selection and the reorganized final list.

Domain	Subdomains	
	Initial	Final^a^
Chronic respiratory disease	Symptoms	Symptoms
Concomitant diseases	Food allergy Rhinitis	Food allergy Rhinitis Respiratory infections Sleep disorders
Exposure to external agents	Allergens Occupational hazards Smoking habits Atmospheric pollution	Allergens Occupational hazards Smoking habits
Nonpharmacological therapies	Breathing exercises and airway clearance techniques Physical activity and exercise Oxygen therapy and ventilatory support	Breathing exercises and airway clearance techniques Physical activity and exercise
Pharmacological therapies	Adherence and inhaler technique Devices and active principles Vaccinations	Adherence and inhaler technique Devices and active principles Vaccinations
Other	N/A^b^	Anxiety, depression, and stress Nutrition

^a^Only subdomains with at least one implemented recommendation are included in this list.

^b^N/A: not applicable.

**Table 2:** Sources of recommendations for the knowledge base.

Clinical area and scope		Authorship	Document title	Source document reference
**Asthma**				
	International	GINA^a^	GINA. Global strategy for asthma management and prevention	GINA 2018 [24]
	National (Portugal)	DGS^b^	NOC^c^ 006/2018 of February 26, 2018: monitoring and treatment to control asthma in children, adolescents, and adults	NOC 2018 [25]
	National (United Kingdom)	NICE^d^	Asthma: diagnosis, monitoring, and chronic asthma management	NICE 2017 [26]
	National (United Kingdom)	BTS^e^ and SIGN^f^	SIGN 153—British guideline on the management of asthma	BTS 2016 [27]
	National (Portugal)	DGS	PNDR^g^, 2012-2016: good practices and guidelines for the control of asthma in adults and children	PNDR 2013 [28]
	Other	Walters et al [29]	Long-acting beta2-agonists in asthma: an overview of Cochrane systematic reviews	Walters et al [29]
**Asthma and pregnancy**				
	National (United States)	National Heart, Lung, and Blood Institute; NAEPP^h^ Asthma and Pregnancy Working Group	Managing Asthma During Pregnancy: Recommendations for Pharmacologic Treatment—2004 Update	NAEPP 2004 [30]
**COPD^i^**				
	International	GOLD^j^	GOLD. Global strategy for the diagnosis, management, and prevention of chronic obstructive pulmonary disease	GOLD 2018 [23]
	National (Australia and New Zealand)	Lung Foundation Australia and the Thoracic Society of Australia and New Zealand	The COPD-X^k^ Plan: Australian and New Zealand Guidelines for the Management of Chronic Obstructive Pulmonary Disease	COPD-X 2018 [31]
	National (United Kingdom)	NICE	Chronic obstructive pulmonary disease in over 16s: diagnosis and management	NICE 2018 [32]
	National (Portugal)	DGS	NOC 028/2011 updated on September 10, 2013: diagnosis and treatment of chronic obstructive pulmonary disease	NOC 2013 [33]
**COPD exacerbations**				
	International	ACCP^l^ and CTS^m^	Prevention of Acute Exacerbations of COPD: American College of Chest Physicians and Canadian Thoracic Society Guideline	ACCP and CTS 2015 [34]
**Comorbidity—allergic rhinitis**				
	International	ARIA^n^	ARIA guidelines: 2010 revision	ARIA 2010 [35]
	International	ARIA in collaboration with the World Health Organization	ARIA	ARIA 2001 [36]
**Comorbidity—OSA^o^**				
	National (Portugal)	DGS	Guidance 022/2014 updated on November 28, 2016: Follow-up in Primary Health Care of Patients With Obstructive Sleep Apnea Syndrome Under Continuous Positive Pressure Therapy	DGS 2016 [37]^p^
	National (United States)	ACP^q^	Management of Obstructive Sleep Apnea in Adults: A Clinical Practice Guideline From the American College of Physicians	ACP 2013 [38]
	Other	Kribbs et al [39]	Objective measurement of patterns of nasal CPAP^r^ use by patients with obstructive sleep apnea	Kribbs et al [39]^p^
**Comorbidity—respiratory infections**				
	National (Portugal)	DGS	NOC 045/2011 of December 26, 2011: antibiotic therapy in community-acquired pneumonia in immunocompetent adults	NOC 2011 [40]
**Breathing exercises**				
	National (United Kingdom)	BTS	BTS guideline on pulmonary rehabilitation in adults: accredited by NICE	BTS 2013 [41]
	National (United Kingdom)	BTS and ACPRC^s^	Guidelines for the physiotherapy management of the adult, medical, spontaneously breathing patient	BTS and ACPRC 2009 [42]
**Oxygen therapy and ventilatory support**				
	National (Portugal)	DGS	NOC 022/2011 updated on September 11, 2015—Home respiratory care: prescription of ventilotherapy and other equipment	NOC 2015 [43]^p^
	National (United Kingdom)	BTS	BTS Guidelines for Home Oxygen Use in Adults	BTS 2015 [44]^p^
**Adherence to treatment**				
	National (United Kingdom)	NICE	Medicine adherence: involving patients in decisions about prescribed medicines and supporting adherence	NICE 2009 [45]
**PA^t^ and exercise**				
	National (United States)	US Department of Health and Human Services	PA Guidelines for Americans, second edition	PA 2018 [46]
	National (United States)	ACSM^u^	ACSM position stand—quantity and quality of exercise for developing and maintaining cardiorespiratory, musculoskeletal, and neuromotor fitness in apparently healthy adults: guidance for prescribing exercise	ACSM 2011 [47]
	National (United States)	ACSM	ACSM position stand—exercise and physical activity for older adults	ACSM 2009 [48]
	National (the Netherlands)	KNGF^v^	Clinical practice guideline for physical therapy in patients with COPD	KNGF 2008 [49]
**Comorbidity—osteoporosis**				
	National (United States)	ACR^w^	Recommendations for the prevention and treatment of glucocorticoid-induced osteoporosis	ACR 2010 [50]

^a^GINA: Global Initiative for Asthma.

^b^DGS: Directorate-General of Health*.*

^c^NOC: Clinical Practice Guideline.

^d^NICE: National Institute for Health and Care Excellence.

^e^BTS: British Thoracic Society.

^f^SIGN: Scottish Intercollegiate Guidelines Network.

^g^PNDR: National Programme for Respiratory Diseases.

^h^NAEPP: National Asthma Education and Prevention Program.

^i^COPD: chronic obstructive pulmonary disease.

^j^GOLD: Global Initiative for Chronic Obstructive Lung Disease.

^k^COPD-X: Australian and New Zealand guidelines for the management of chronic obstructive pulmonary disease.

^l^ACCP: American College of Chest Physicians.

^m^CTS: Canadian Thoracic Society.

^n^ARIA: Allergic Rhinitis and its Impact on Asthma.

^o^OSA: obstructive sleep apnea.

^p^No recommendations from this source included in the final selection.

^q^ACP: American College of Physicians.

^r^CPAP: continuous positive airway pressure.

^s^ACPRC: Association of Chartered Physiotherapists in Respiratory Care.

^t^PA: physical activity.

^u^ACSM: American College of Sports Medicine.

^v^KNGF: The Royal Dutch Society for Physiotherapy.

^w^ACR: American College of Rheumatology.

### Structure of Data Collection

The knowledge base was structured according to the 6 defined domains. Considering its future application in a health coaching component, 2 in-person meetings were held between health care and IT professionals within 3 weeks. The final structure was approved through consensus.

For each recommendation, the following characteristics were defined as relevant and extracted: (1) type of source document, (2) original text recommendation with page number, (3) recommendation ID, (4) source document reference, (5) original level of evidence, (6) harmonized level of evidence (HLE), (7) domain, (8) subdomain, and (9) target group of patients.

The HLE was created through consensus to convey the diversity of the classification systems of the level of evidence and grades of recommendation of each source document (Table S1 in Multimedia Appendix 1 [23-25,27,31,33-35,38,41-44,47-50]). This HLE followed the GINA [24] and GOLD [23] classifications.

All characteristics were considered mandatory except for the original level of evidence and HLE. Only the levels of evidence explicitly reported in the original data sources were considered; when no level of evidence was reported in the source document, both the original and HLE were registered as missing.

### Identification of Recommendations: From Initial Selection to Prioritization

According to the professional background and areas of scientific interest, each health care professional thoroughly reviewed relevant data sources and made an initial identification of pertinent recommendations. To be considered for inclusion, the recommendations should be (1) applicable to individuals with CORDs or to prevent CORDs, (2) directed to patient self-management (eg, recommendations regarding drug therapy were only included if they could be given to the patient in compliance with their action plan), (3) targeting adults (recommendations that were exclusively applicable to children were not included), and (4) within the scope of the selected domains and subdomains (Table 1).

Along with this initial identification, a priority classification was issued for each selected recommendation based on (1) the HLE; (2) the scope of the source document (international recommendations were preferred); (3) the entity that issued the source document; (4) the operability of the recommendation (eg, type of data that would be needed to implement it in a personalized way); and (5) health care professionals’ perceptions of the relevance, potential impact, and reach of the recommendation. This classification was conducted on a scale of 1 (highest priority) to 3 (lowest priority) in 2 phases: first individually by each health care professional and then reviewed by the group. Disagreements were resolved through consensus among the members of the multidisciplinary team, and if deemed necessary, the priorities were reclassified.

Finally, a further selection refinement was made in 2 additional in-person team meetings intending to maintain a balance between the scope of this clinical knowledge base and the feasibility of its implementation, use, and update. When several recommendations overlapped, those assigned a lower priority level were excluded. Moreover, recommendations were also excluded if they were only applicable to a small number of individuals (eg, just for patients on long-term oxygen therapy or with a very strict combination of characteristics) or if their personalized implementation could only be achieved with a high effort (eg, with the need to collect a lot of data) or with the use of specific sensors that are not included in most smartphones (eg, oximetry for overnight monitoring).

### Description of the Recommendations

A total of 667 recommendations from 17 subdomains were identified by the health care professionals; 35.4% (n=236) were considered priority 1, and over one-third (n=250, 37.5%) concerned disease symptoms (Table 3 and Table S2 in Multimedia Appendix 2). After prioritization and selection refinement, 53.7% (358/667) of the recommendations were selected for implementation. A total of 38.8% (139/358) were related to pharmacological or nonpharmacological therapies for CORDs, and 30.4% (109/358) were related to CORD symptoms. Most were retrieved from international (169/358, 47.2%) or national (154/358, 43%) guidelines for respiratory diseases, but 9.8% (35/358) came from nonrespiratory guidelines or other types of sources. In total, 22.9% (82/358) were classified as HLE A (highest); nevertheless, in 44.4% (159/358), the evidence level was either not reported or uncertain in the original data source.

**Table 3:** Description of the selected recommendations considering all those that were identified in the initial selection and those that were selected for further implementation.

				Initial selection (n=667), n (%)		Implemented (n=358), n (%)		Implemented, n (% of the initial selection)
**Domain and subdomain**								
	**Chronic respiratory disease**			250 (37.5)		109 (30.4)		109 (43.6)
		Symptoms	250 (37.5)		109 (30.4)		109 (43.6)	
	**Concomitant diseases**			62 (9.3)		35 (9.8)		35 (56.5)
		Food allergy	5 (0.7)		3 (0.8)		3 (60)	
		Rhinitis	15 (2.2)		7 (2)		7 (46.7)	
		Respiratory infections	23 (3.4)		18 (5)		18 (78.3)	
		Sleep disorders	19 (2.8)		7 (2)		7 (36.8)	
	**Exposure to external agents**			70 (10.5)		50 (14)		50 (71.4)
		Allergens	49 (7.3)		30 (8.4)		30 (61.2)	
		Occupational hazards	5 (0.7)		4 (1.1)		4 (80)	
		Smoking habits	16 (2.4)		16 (4.5)		16 (100)	
		Atmospheric pollution	0 (0)		0 (0)		0 (0)	
	**Nonpharmacological therapies**			87 (13)		58 (16.2)		58 (66.7)
		Breathing exercises and airway clearance techniques	19 (2.8)		18 (5)		18 (94.7)	
		Physical activity and exercise	42 (6.3)		40 (11.2)		40 (95.2)	
		Oxygen therapy and ventilatory support	26 (3.9)		0 (0)		0 (0)	
	**Pharmacological therapies**			161 (24.1)		81 (22.6)		81 (50.3)
		Adherence and inhaler technique	93 (13.9)		39 (10.9)		39 (41.9)	
		Devices and active principles	50 (7.5)		31 (8.7)		31 (62)	
		Vaccinations	18 (2.7)		11 (3.1)		11 (61.1)	
	**Others**			37 (5.5)		25 (7)		25 (67.6)
		Anxiety, depression, and stress	17 (2.5)		9 (2.5)		9 (52.9)	
		Nutrition	20 (3)		16 (4.5)		16 (80)	
**Target group of patients**								
	All			22 (3.3)		6 (1.7)		6 (27.3)
	Asthma			301 (45.1)		162 (45.3)		162 (53.8)
	COPD^a^			265 (39.7)		145 (40.5)		145 (54.7)
	Healthy			55 (8.2)		42 (11.7)		42 (76.4)
	Long-term oxygen therapy			16 (2.4)		0 (0)		0 (0)
	Rhinitis			8 (1.2)		3 (0.8)		3 (37.5)
**Scope of the source document**								
	Respiratory—international			315 (47.2)		169 (47.2)		169 (53.7)
	**Respiratory—national**			311 (46.6)		154 (43)		154 (49.5)
		Australia and New Zealand	75 (11.2)		47 (13.1)		47 (62.7)	
		Portugal	45 (6.7)		27 (7.5)		27 (60)	
		United Kingdom	185 (27.7)		76 (21.2)		76 (41.1)	
		United States	6 (0.9)		4 (1.1)		4 (66.7)	
	**Nonrespiratory—national**			39 (5.8)		34 (9.5)		34 (87.2)
		The Netherlands	7 (1)		6 (1.7)		6 (85.7)	
		United States	32 (4.8)		28 (7.8)		28 (87.5)	
	Others			2 (0.3)		1 (0.3)		1 (50)
**HLE^b^**								
	A			110 (16.5)		82 (22.9)		82 (74.5)
	B			67 (10)		42 (11.7)		42 (62.7)
	C			68 (10.2)		36 (10.1)		36 (52.9)
	D			64 (9.6)		39 (10.9)		39 (60.9)
	Not reported or doubtful^c^			358 (53.7)		159 (44.4)		159 (44.4)
**Priority of the recommendation**								
	1 (highest)			236 (35.4)		165 (46.1)		165 (69.9)
	2			279 (41.8)		142 (39.7)		142 (50.9)
	3 (lowest)			152 (22.8)		51 (14.2)		51 (33.6)

^a^COPD: chronic obstructive pulmonary disease.

^b^HLE: harmonized level of evidence.

^c^In total, 3 recommendations had 2 different evidence levels reported within the same source document (A or not reported: n=1, 33%; B or D: n=1, 33%; C or D: n=1, 33%).

## Operationalization and Implementation of the Knowledge Base

### Overview

This knowledge base of recommendations for CORD self-management was developed keeping in mind its future deployment in mHealth systems, namely, a health coaching component within the AIRDOC ecosystem. Therefore, it should provide a proper and adequate basis for the delivery of decision support at the time and location of decision-making and provide actionable recommendations. To turn the original recommendations (directly retrieved from the source documents) into real-time actionable personalized recommendations, there was a need to identify relevant variables and build logical rules that could trigger each recommendation to the right patient at the right time.

### Creation of Logical Rules to Personalize Recommendations

The relevant patient or environmental data that would be required to build logical rules and personalized recommendations, as indicated or suggested in the original recommendations, were listed as individual variables. For each variable, a definition and potential data sources were proposed (eg, direct questions to the user, smartphone sensors, wearables, and external databases accessed via internet connection). In cases in which the variable of interest could be broken down into multiple elementary variables (eg, asthma control could be defined based on the Control of Allergic Rhinitis and Asthma Test score that was computed as the sum of its 10 questions [51]), all the variables that would be needed to compute it were identified and defined (eg, not only the total Control of Allergic Rhinitis and Asthma Test score, which was directly used in most rules, but also each of the questions that are part of the questionnaire were included in the variable list). When similar or equal input variables were identified for different recommendations, they were harmonized by the research team to avoid duplication and keep the variable list as uniform as possible. Whenever available, previously existing standardized questionnaires and health IT standards (eg, openEHR [52] and Fast Healthcare Interoperability Resources; FHIR [53,54]) were used for guiding the variable definition and its structure and intrinsic attributes.

Logical rules were built using the identified variables so that each recommendation was only triggered in the presence of a set of relevant characteristics and provided to those individuals to which it applied (using IF-THEN statements). When a rule required the combination of multiple variables, they were connected via Boolean logical operators. In the case of recommendations that could be applied to different sets of patients, several rules were created, each one targeting one group of patients (eg, a rule for patients with asthma and another for those with COPD). When the original recommendation missed specific or quantitative information that was relevant to creating the rule (eg, specific tool and respective cutoff to define medication adherence), the missing aspects were decided through consensus accounting for the available literature, the usual clinical practice, and ease of implementation. Moreover, limits of changes in longitudinal variables were established through consensus either in absolute or relative units (eg, a significant weight loss was defined as losing at least 10% of the body weight in ≤6 mo).

**Figure 3:**
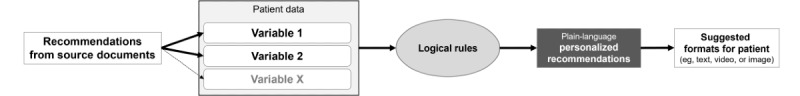
Pathway from original recommendations to implementation as rule-based, actionable, plain-language personalized recommendations that can be delivered to patients using different formats.

Finally, each recommendation was adapted to plain language. Suggestions regarding possible ways of delivering the recommendation to the user (eg, text, image, or video) were also discussed and recorded. The pathway from the original recommendation to the implementation is summarized in Figure 3.

As an example, an original recommendation from GINA [24] stating that “(for patients with asthma) Encourage smoking cessation by patient/family; provide advice and resources” could be triggered by the presence of asthma and currently smoking at least one cigarette per day (logical condition: “self-reported asthma”=TRUE AND “current number of cigarettes/day”≥1). The plain-language personalized recommendation was “Smoking cessation is advised,” and it could be provided using text, videos, or images or graphs focusing on the immediate and long-term advantages of quitting smoking and complemented with links to additional resources encouraging smoking cessation.

### Assignment of a Priority Level to Personalized Recommendations

As it was expected that several personalized recommendations could apply to the same individual at a single time point, it was considered necessary to assign each recommendation a priority level that could be used to support the selection of which recommendations to trigger first. This classification was based on the priority level assigned to the original recommendation and refined via the health care professionals’ perceptions of the relevance and impact of the personalized recommendation on patient outcomes. This was conducted considering the stratification by clinical subdomains and the target disease groups. A scale of 1 (highest priority) to 5 (lowest priority) was used, and the same 2-phase methodology described in the prioritization of the original recommendations was implemented.

### Implementation in the Decision Support System

**Figure 4:**
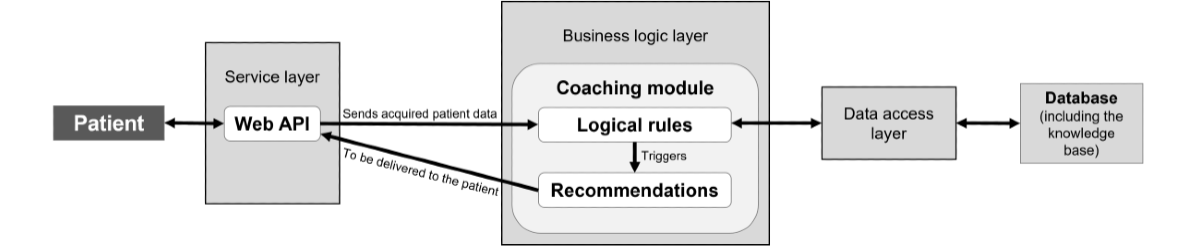
Architecture of the intelligent individualized coaching component to be integrated into the clinical decision support system. Adapted from Vieira et al [55]. API: application programming interface.

The knowledge base (including the logical rules, actionable personalized recommendations, and source recommendations supporting them) was implemented in an intelligent individualized coaching component. The architecture of this component has been published elsewhere [55], and it is briefly described in this section and summarized in Figure 4. This component was built to be integrated into mHealth support systems and, therefore, includes 3 layers: service layer, business logic layer, and data access layer.

The service layer serves as a gateway between the coaching module and external services. This layer contains the web application programming interface (API), which provides a set of services to acquire information (self-reported by the patient, automatically acquired by the smartphone, or from other external sources) and deliver the personalized recommendation to the patient along with a path to the source recommendation supporting it.

The business logic layer contains the coaching module, which processes the information received by the web API according to the defined logical rules and sends the generated personalized recommendations to the web API to be delivered to the patient. When several personalized recommendations are triggered by the available patient characteristics, the order in which they are presented is primarily defined by the assigned priority; when recommendations with similar priority can be delivered, the order in which they are sent to the patient will be randomly defined by the system. This coaching module was developed in Drools [56], which is the most widely used open-source rule engine framework for the Java programming language [57] and provides the mechanisms for easy rule addition, removal, and editing.

The data access layer serves as an intermediate layer between the business logic layer and the database. This service receives requests from the business logic layer about whether to read, insert, update, or delete information available in the database. The database contains information regarding the patient’s clinical data, variables associated with recommendations, and the history of provided recommendations. It also contains the knowledge base of recommendations, including the assigned priority level.

### Description of the Implemented Logical Rules and Personalized Recommendations

Overall, 405 logical rules (personalized recommendations) were created based on the 358 selected recommendations (mean 1.1, SD 0.6; maximum of 4 rules per recommendation); all were implemented in the intelligent individualized coaching component.

To create these rules, 116 variables were defined: 31 (26.7%) were used in only 1 rule, 35 (30.2%) were used in 2 to 3 rules, 30 (25.9%) were used in 4 to 10 rules, and 20 (17.2%) were used in >10 rules. The most frequently used variables are listed in Table S3 in Multimedia Appendix 3. The mean number of variables that were used to trigger a personalized recommendation was 3.5 (SD 1.8), ranging between 1 (34/405, 8.4%) and 9 (3/405, 0.7%).

There were 208 unique logical conditions (ie, combinations of variables and logical operators excluding the recommendation itself) triggering the 405 personalized recommendations. Most logical conditions (156/208, 75%) triggered a single recommendation, 21.2% (44/208) triggered 2 or 3 recommendations, and 3.8% (8/208) triggered ≥4 recommendations (maximum of 62; Table S3 in Multimedia Appendix 3). The conditions that triggered a higher number of recommendations were mostly based on general individual characteristics (eg, age and disease status; Table S3 in Multimedia Appendix 3).

More than one-third (149/405, 36.8%) of the personalized recommendations were considered to be of high priority (level ≥2 out of 5; Table S3 in Multimedia Appendix 3).

To pretest the applicability of the knowledge base, a typical use case in clinical practice—a young adult male with uncontrolled asthma and persistent rhinitis—was simulated. A full description of the patient characteristics is provided in Table S4 in Multimedia Appendix 4. Overall, 78 recommendations were generated using the available information. The characteristics that triggered the highest number of recommendations were the presence of self-reported asthma, sensitization to at least one (indoor) allergen, and prescribed medication (Table S4 in Multimedia Appendix 4). It is important to note that being a nonsmoker, having no exposure to secondhand tobacco smoke, using a low dose of inhaled corticosteroids, having no hospitalizations or emergency department visits in the previous year, and presenting a percentage of predicted forced expiratory volume in the first second within the normal range were not used to trigger any recommendation. This suggests that recommendations were created mostly based on the presence of a “negative” characteristic to support some kind of behavior change and not to reinforce already existing “positive” features.

## Initial Validation of the First Version of the Operationalized Knowledge Base

The initial validation included two steps: (1) an internal evaluation by the development team using clinical data from a previous study with patients with CORDs to assess the clinical adequacy of the triggered personalized recommendations and identify possible implementation issues and (2) an external assessment of physicians’ opinions on the appropriateness of the personalized recommendations assigned the highest priority.

### Internal Validation Using a Clinical Data Set

To assess the clinical adequacy of the recommendations in the knowledge base, they were tested using anonymized data from a nationwide cross-sectional study conducted in the Portuguese general population, the Control and Burden of Asthma and Rhinitis (ICAR) study [58]. This study enrolled 728 adults (aged ≥18 y) with and without self‐reported asthma or rhinitis; individuals with COPD were not specifically targeted. For each participant, the available data included sociodemographic characteristics, medical diagnoses (respiratory diseases and comorbidities), symptoms (focused on respiratory diseases), exposure to risk factors (eg, smoking), current treatment, exacerbations, use of health care services, skin prick tests results, spirometry parameters, and fraction of exhaled nitric oxide. A description of ICAR participants is provided in Table S5 in Multimedia Appendix 5.

The available variables from ICAR were mapped to those needed to feed the logical conditions and trigger personalized recommendations. Mapping was conducted by 2 members of the knowledge base development team who were also involved in the ICAR study. ICAR data were only used to feed those variables that had a similar definition in both data structures; when necessary, they were recoded (eg, inhaled corticosteroid dose was recoded from continuous to categorical), and new variables were computed (eg, the presence of at least one risk factor for asthma exacerbation was calculated based on several ICAR variables reporting specific risk factors), always respecting the assigned definitions. Neither imputation of missing values nor random data attribution was performed to fill in the missing data. Variables that relied on longitudinal data (eg, trend in asthma control over 3 mo) could not be completed using ICAR data. Overall, these data could be used to feed 63.8% (74/116) of the variables that would be required to test the entire range of personalized recommendations.

Considering ICAR participants, a total of 26,612 personalized recommendations were generated, with a median of 23 (IQR 19-25) and a maximum of 176 recommendations per participant. In total, 24.3% (177/728) of the participants had >25 recommendations; the characteristics associated with triggering a higher number of recommendations are described in Table S5 in Multimedia Appendix 5. No healthy individuals or those with another respiratory disease (other than asthma, COPD, or rhinitis or rhinosinusitis) had >25 recommendations.

Overall, 65.2% (264/405) of the personalized recommendations were triggered at least once (median 11, IQR 6-156 times; maximum of 649 times). Of the 405 personalized recommendations, 141 (34.8%) were not triggered, of which 109 (77.3%) needed an unavailable variable, 5 (3.5%) strayed from the knowledge base scope and targeted pediatric patients (and were excluded), 4 (2.8%) had logical errors in the rule that made them not triggerable (and were afterward corrected), and 23 (16.3%) were not used as no ICAR participants met all the characteristics that would trigger those recommendations.

To assess the clinical adequacy of the personalized recommendations, a sample of 9.9% (72/728) of ICAR participants was randomly selected, and each patient’s data (including clinical characteristics and triggered recommendations) were reviewed by the knowledge base development team. Each triggered personalized recommendation was classified as correct or incorrect or in need of improvement considering the specific patient characteristics and the scope of the original recommendation. Suggestions were issued for recommendations classified as incorrect or in need of improvement.

This patient sample generated a total of 3049 personalized recommendations, with a median of 24 (IQR 20-80) and a maximum of 176 recommendations per participant. Overall, 56.8% (230/405) of the personalized recommendations were triggered at least once (median 5, IQR 1-21 times; maximum of 64 times).

A total of 5.84% (178/3049) of the analyzed recommendations were classified as needing improvement. Of the 230 personalized recommendations that were triggered in this sample, 48 (20.9%) were reported as being not completely correct. Most issues (34/48, 71%) were related to inadequate personalization with the need to include additional variables in the logical condition to make it more specific. An example is the original recommendation “There is insufficient evidence to support one stress-reduction strategy over another, but relaxation strategies and breathing exercises may be helpful” from GINA [24], which originally only required the presence of an asthma diagnosis to be triggered. In this review process, it was suggested to include the presence of anxiety (assessed using the anxiety subscale of the Hospital Anxiety and Depression Scale [59]) as an additional necessary condition to trigger this recommendation. Some additional suggestions were related to the need to avoid overlapping recommendations (7/48, 15%) and to increase the adequacy of the recommendation to the patient (2/48, 4%). However, none of the recommendations reported as not completely correct were identified as posing any risk to the patients for whom they were generated.

The implementation of the knowledge base in the intelligent individualized coaching component was also tested. Overall, 26,135 recommendations were triggered by the computerized support system using ICAR data; however, 540 recommendations that should have been actioned were not generated. Moreover, 63 nontriggerable personalized recommendations were generated. Issues concerning implementation or data presentation in 12 variables were identified as causing these differences. Most issues (n=8) were in numerical variables whose answers were not adequately formatted or read as numbers; the others were in categorical variables that were input with minor differences compared with those expected by the system (eg, initial capital letter). These issues prevented the correct activation of 17 logical conditions in a total of 21 personalized recommendations. Nevertheless, using the intelligent support system, 97.97% (26,072/26,612) of the personalized recommendations were activated according to the respective rules, and all the identified issues were easy to resolve.

### External Physicians’ Validation of the Appropriateness of Level-1 Priority Recommendations

The 47 personalized recommendations that were classified as having the highest priority level (priority level 1; Table S3 in Multimedia Appendix 3) were independently reviewed by 4 physicians with different backgrounds and who were not involved in the knowledge base development. They were asked to assess whether the personalized recommendations (including their triggering logical condition) were a correct implementation of the original recommendation and whether they would be appropriate for end users (ie, patients with CORDs). Moreover, they were asked to provide suggestions for improvement when applicable.

All recommendations were considered adequate to patients by at least one of the reviewing physicians. Nevertheless, 4% (2/47) of the recommendations were identified as not being appropriate to patients by at least 2 reviewers. These 2 recommendations were related to data reporting and documentation of smoking history and the need for additional clinical assessment in smokers. In fact, these source recommendations were not clearly directed to patients, and the initial implementation was not optimized for this target; nevertheless, it was possible to define a different implementation strategy that may allow for the use of these recommendations to promote patient engagement with data collection, reinforcing the relevance of these specific aspects and supporting the need to notify patients when these data are missing.

In 68% (32/47) of the recommendations, the reviewing physicians provided suggestions to improve the practical implementation (eg, use of visual tools that were not previously indicated) and the text of the personalized recommendation to be sent to the patient. In 26% (12/47) of the recommendations, the reviewing physicians suggested changes to the logical conditions to increase personalization. Moreover, 30% (14/47) of the recommendations were identified as overlapping with others (usually from different sources), and suggestions were issued regarding joining or dismissing some of these recommendations. Moreover, a few suggestions were made concerning the possible application of some of the recommendations to other patient groups not specifically targeted in the original sources (eg, a recommendation regarding medication adherence from Australian and New Zealand guidelines for the management of COPD 2018 [31] that originally targeted patients with COPD was suggested as applicable to patients with asthma).

Only 1 additional recommendation was suggested; this recommendation was based on an update to the GINA guidelines [60] and will be included in the next version of the knowledge base.

On the basis of these initial assessments (summarized in Table 4), several issues were identified and were or will be resolved in future updates to the knowledge base. Testing the knowledge base using anonymized clinical data available from a previous study provided valuable information and should be repeated following future changes.

**Table 4:** Summary of performed assessments and their major findings.

Type of assessment		Aim	Major results	Identified issues and aspects in need of improvement	Solutions (implemented or TBI^a^)
Use case^b^		Test the applicability of the knowledge base with a typical clinical case (asthma and rhinitis)	A total of 78 recommendations were generated. The highest number of recommendations was triggered by the presence of self-reported asthma, being sensitized to ≥1 (indoor) allergens, and having prescribed medication.	There were no major issues. Recommendations were created mostly based on the presence of a “negative” characteristic to support some kind of behavior change (vs reinforcing “positive” features).	Include additional recommendations that positively reinforce healthy behaviors (TBI).
**Internal assessment using clinical data^b,c^**		General description of the triggered recommendations	A total of 26,612 recommendations were generated for 728 individuals (maximum of 176/participant). One-quarter had >25 recommendations. A total of 264 (65%) recommendations were triggered ≥1 time.	A total of 141 (35%) recommendations were not triggered with this data set: A total of 77% needed an unavailable variable. A total of 4% strayed from the knowledge base scope (targeted pediatric patients). A total of 3% had logical errors that made them not triggerable. A total of 16% were not triggered because of lack of ICAR^d^ patients with all the necessary characteristics.	Rules errors were corrected. Rules out of the knowledge base scope were excluded. Conduct additional validation, including patients with other characteristics (TBI). Conduct targeted studies collecting all cross-sectional and longitudinal data that are needed to feed the rules (TBI).
	Clinical adequacy^b^	Assess the clinical adequacy of the triggered personalized recommendations in a random sample of 72 patients	A total of 3049 recommendations were generated. A total of 230 (57%) recommendations were triggered ≥1 time.	A total of 20.9% (48/230) of the triggered recommendations were reported as being not completely correct: A total of 71% had issues related to inadequate personalization. A total of 15% were overlapping recommendations. A total of 4% were considered more adequate for health care professionals than for patients.	Review and reassess the recommendations in need of improvement (TBI). Implement an additional refinement process to foster the identification of “clusters” of similar or overlapping recommendations (TBI).
	Implementation^e^	Identify possible implementation issues	A total of 26,135 recommendations were triggered for 728 individuals. A total of 98% of the personalized recommendations were activated according to the respective rules.	A total of 540 recommendations that should have been actioned were not generated. A total of 63 nontriggerable personalized recommendations were generated. Issues in 12 variables were identified (concerning implementation or data presentation), with an impact on 17 logical conditions and 21 personalized recommendations.	All identified issues were corrected.
External physician assessment^f^		Assess (using 47 personalized recommendations with the highest priority) whether the personalized recommendations correctly implemented the original recommendation and whether they were appropriate for the intended users (patients)	All recommendations were considered adequate for patients by at least one of the reviewers.	A total of 32 (68%) of the recommendations would benefit from improved practical implementation. A total of 12 (26%) recommendations would benefit from increased personalization. A total of 14 (31%) recommendations were considered as overlapping with others.	Same as in the assessment of clinical adequacy (TBI)

^a^TBI: to be implemented.

^b^Conducted by the development team.

^c^Data from a previous study with patients with chronic obstructive respiratory diseases, the Control and Burden of Asthma and Rhinitis study.

^d^ICAR: Control and Burden of Asthma and Rhinitis.

^e^Conducted using the intelligent individualized support system and critically reviewed by the development team.

^f^Conducted by 4 physicians not involved in the knowledge base development.

In addition to these already identified issues, with evolving knowledge, the evidence-based guidelines and protocols are constantly evolving and expanding, raising the need for regular knowledge base updates. Keeping the knowledge base up-to-date is an utterly important task that prevents loss of relevance and outdated recommendations being issued and promotes an efficient implementation of the most recent clinical guidelines.

## Strategy to Update the Knowledge Base

The update strategy was defined by the multidisciplinary development team. The structure of the knowledge base and the requirements for its implementation in the intelligent individualized coaching system (which included the need for a rules editor) were developed to allow for the possibility of periodic updates. These updates can occur as described in Textbox 1.

The update of the knowledge base should be done regularly at defined intervals [61]. Considering the recommended periodicity for clinical practice guideline updates (2-3 years) [62,63] and the usual time frame to update the most relevant guidelines for CORD management (every year for GINA [24] and GOLD [23]), an update every 1 or 2 years is proposed. Nevertheless, minor updates to resolve inconsistencies in the logical conditions or personalized recommendations can be done constantly whenever they are identified as part of a continuous improvement strategy. In fact, once the necessary updates are defined, with the relevant variables identified, the logical rules built, and the plain-language personalized recommendations set, the rules editor allows for prompt implementation in the intelligent individualized coaching component.

Although it is easy to implement, this update process should be carried out in a controlled manner, with a limited number of persons allowed as editors and replicating the internal validation upon each major update. Moreover, changes from one version to the other must be identified and adequately registered, keeping an ongoing, up-to-date audit trail. Furthermore, adequate training should be provided to new staff joining the knowledge base development team to ensure that consistency with the preexisting system or knowledge base is maintained. As the update process is simple, how-to videos and having team members capable of answering questions or providing direct training can help maintain the quality. However, there will be ongoing costs for system updates that are required to keep pace with knowledge.

The ability to efficiently update the knowledge base to take the new evidence into account soon after it is issued and apply it seamlessly in the clinical context might have a strong impact on adoption. By doing so in the context of patient-centered care through a knowledge base that will be implemented in an intelligent CDSS, a rapid dissemination of knowledge into clinical practice is expected.

**When to update the knowledge base.**

**Knowledge base updates can be conducted in 3 general cases:**

- To update an existing clinical recommendation
- To include a new recommendation relevant to current clinical practice
- To exclude an outdated recommendation

**A new recommendation can be introduced in the following cases:**

- Because of evolving information on the domains already included in the knowledge base
- To cover additional domains or subdomains that present increasing relevance to chronic obstructive respiratory disease management, widening the scope of the knowledge base
- To take advantage of improved data collection technologies that might create the conditions to apply already existing recommendations that were not implemented because of difficulties in adequately or easily collecting relevant trigger data (eg, because of lack of smartphone sensors)

## Discussion

### Critical Overview of Major Findings

A multidisciplinary framework was used in the development of this knowledge base to ensure that an individualized CDSS for patients with CORDs is based on the best available evidence at any time in the future. We described the development process showing how a multidisciplinary team of health care, data sciences, and IT professionals translated existing disease guidelines into personalized plain-language patient recommendations to be used as part of mHealth decision support systems. The initial assessment processes were conducted using anonymized clinical data from a previous study with patients with CORD and complemented with the opinion on the relevance of the selected recommendations by physicians who were not involved in the knowledge base development. Multiple aspects needing improvement have already been identified and will be integrated into future versions of the knowledge base as part of a continuous improvement process; a periodic update, with a predicted 1- to 2-year time frame, will be implemented to account for evolving knowledge. This will be done by taking advantage of the implemented rules editor, which allows for easy and prompt implementation of the necessary changes. We described this process for the self-management of patients with CORDs, but it may be used as a guide to build other knowledge bases to support patients with different diseases.

Only a few studies have described the process of knowledge base development [17,19,64-67], and most have done so as a small section within the construction of a specific CDSS [19,64-66]. To our knowledge, only 1 study has provided a full description [67], and another has provided a partial description [19] of a knowledge base development within the scope of respiratory diseases. Shegog et al [67] used a pragmatic methodology including a traditional inductive approach to understanding clinical experts’ decision processes for several asthma domains (ie, severity and control as well as self-management) followed by a deductive approach to validate a conceptual framework for asthma self-management. Knowledge acquisition was based on expert interviews (focused on 10 case vignettes) and work groups and used theoretical and empirical evidence on asthma and other chronic diseases as part of the deductive approach [67]. Velickovski et al [19] described the knowledge base development as part of a full CDSS for the preventive management of patients with COPD. Knowledge acquisition was based on clinical guidelines (only GOLD was explicitly referred to) interpreted by respiratory specialists and defined as rules or algorithms. It focused on COPD detection and diagnosis, spirometry quality control support, and patient stratification [19]. Both studies aimed to develop knowledge bases to support clinicians during health care provision. Conversely, our knowledge base was built to support patient self-management, focusing on recommendations that can be directly applied to patients. We used a guideline-based knowledge acquisition similar to that used by Velickovski et al [19]. Nevertheless, we considered a wider scope regarding not only the clinical aspects included but also the guidelines that were reviewed. This process is different from that used by Shegog et al [67] and has the advantage of using knowledge that comes directly from evidence-based disease guidelines, which already include the perspectives of hundreds of experts [68]. Although most guidelines do not specifically consider patient self-management and personalized care, our development framework included a multidisciplinary expert team that adapted these source recommendations to personalized plain-language recommendations that can be issued to patients. Moreover, we fully described our knowledge base development process and framework, allowing this process to be replicated by others.

Building a CDSS knowledge base and keeping it up-to-date requires a tremendous human expert effort, as stated by Aleksovska-Stojkovska et al [17]. Nevertheless, a CDSS can be only as effective as its underlying evidence base [15,21]. Although there are alternatives to knowledge-based CDSSs, the so-called non–knowledge-based CDSSs, they still present important challenges that hinder a more generalized implementation [69,70]. These non–knowledge-based systems still require a data source but use a computer as the central processing unit to learn from historical information through the use of artificial intelligence, machine learning, or statistical pattern recognition [70,71]. The difficulties in understanding the logic behind the produced recommendations, with the CDSS being a black box to the users [70,71], and problems with data availability [69] might be behind the lower-than-expected impact of these non–knowledge-based CDSSs in clinical practice [69]. Nevertheless, most knowledge-based CDSSs are also a black box as the inputs and rationale behind the generated recommendations are not transmitted to the users. Our knowledge base has the advantage of being built keeping a link to the original source recommendation in the intelligent individualized coaching component (ie, the coaching component sends both the plain-language personalized recommendation and the source recommendation supporting it to the user). Moreover, it is possible to also send the rule triggering each specific recommendation so that the user feels more involved in their management, as recently suggested [71].

In a knowledge-based CDSS, rules can be made using literature-based, practice-based, or patient-directed evidence [15,70]. Our knowledge base was developed using guideline-based recommendations that combine the best literature- and practice-based evidence. The GINA [24] and GOLD [23] guidelines, 2 renowned international guidelines supporting the management of patients with CORDs, were used as the major references, and in addition, several other national and international guidelines were selected mostly to complement specific aspects that were not deeply approached in GINA or GOLD. This selection was not based on a systematic review of the published guidelines, which is a limitation of our approach. Nevertheless, we reviewed a broad spectrum of guidelines, and considering the high degree of overlapping between recommendations from different documents and that many national guidelines use GINA or GOLD as a basis to adapt and issue nationally targeted recommendations [72], we believe that including additional national guidelines for patients with CORDs would not bring a significant benefit at the cost of increasing the development workload. Moreover, to develop this knowledge base, a balance between the difficulty of implementation and the overall scale of the covered clinical domains was attempted, and thus, the available evidence within the selected guidelines is not fully covered. In fact, several patient groups have a low number of assigned recommendations, and most target behavior change. Although this was expected considering the aim of the knowledge base [73], it might be relevant to include additional recommendations to reinforce already existing “positive” behaviors [74] and target currently underrepresented groups that might be clinically relevant (eg, patients with obstructive sleep apnea).

The selection of the source documents and original recommendations, as well as the development and writing of the logical conditions and plain-language personalized recommendations, were conducted by a multidisciplinary team including health care professionals with different backgrounds and with academic and clinical experience in the care of patients with CORDs. All the team members were Portuguese, which may have limited our perspective. Our approach was also limited by the lack of direct patient involvement. Nevertheless, both international experts and patients will be part of the team in future updates, further strengthening this process and bringing a broader perspective to the selected recommendations and forms of implementation. To build this knowledge base, the team defined specific inclusion criteria, a consensus prioritization scheme for the selected recommendations, and a common structure and rationale to build the logical conditions and plain-language recommendations. However, although these selections and development choices were based on predefined criteria, it is not possible to completely avoid subjectivity in the decisions that were made. This subjectivity might be related to the results from the initial internal and external assessment processes that showed that approximately one-fourth to two-thirds, respectively, of the recommendations would need improvements before patient testing. Although supported by teamwork and internal revision processes, most logical rules and suggestions for implementation were individually developed and, when reviewed by the team, were supported by the rationale underlying the choices. When evaluated independently from the rationale (both in internal and external assessments), some were perceived as not completely clear and in need of improvement. Nevertheless, in the assessment conducted by external physicians, all the highest-priority recommendations were considered adequate for patients by at least one of the reviewers. These evaluations reinforced the relevance of internal and external validation when developing and implementing rule-based personalized recommendations. Future evaluations of the knowledge base should be also grounded in patient feedback. The inclusion of a feedback feature in the coaching module, in which patients will rank the relevance of that recommendation for their self-management, is being considered.

The use of different sources of recommendations led to a perceived high degree of overlapping between personalized recommendations. This was already expected considering the use of several different data sources targeting similar diseases and was partially addressed when prioritizing the personalized recommendations. Nevertheless, it highlighted that an additional refinement will be needed complementing the already implemented recommendation prioritization. Similar recommendations must be identified, and these “clusters” should be analyzed, with the deletion or redefinition of recommendations that fully overlap and a within-cluster prioritization process. Moreover, additional rules to limit the number of recommendations from the same cluster given to the patient within a specific time frame must also be defined (eg, if a personalized recommendation from a specific cluster is given today, a second recommendation from the same cluster can only be issued after a few days or after a defined number of recommendations).

Furthermore, the knowledge base was not independently validated in real-life situations, nor was it possible to fully validate all the generated rules; thus, the results should be interpreted with caution. Specifically, most rules targeting patients with COPD could not be tested using the available clinical data (ICAR study) and should be tested in future studies. Moreover, the data we used to test the personalized recommendations did not include information regarding one-third of the variables included in the knowledge base. Although this will be addressed in future pilot studies, the presence of missing data to trigger some of the recommendations might hinder their effectiveness. In fact, 15.6% (63/405) of the personalized recommendations required data from ≥6 variables. To minimize a possible low number or lack of recommendations to give to a specific patient, several intelligent strategies are being considered for implementation in the individualized coaching component. When 1 or 2 variables that are needed to trigger a recommendation have no available information, the user will be asked to fill in the missing data. If this strategy fails, case-based reasoning will be used to verify whether there are cases in which recommendations can be generated even though there is no full knowledge about the patient; for example, when one specific recommendation cannot be triggered because of missing data in only one of the required variables, the system will be able to assess the previous uses of that recommendation and the prevalence of the missing characteristic and provide the recommendation if most patients with similar characteristics also received that specific recommendation. As the individualized coaching component is being built to allow for patients’ feedback regarding the adequacy of the issued recommendations, we expect to iteratively refine the recommendations and progressively improve personalization. Nevertheless, this was not tested in this initial validation and should be assessed in future studies.

### Conclusions

We developed a clinical knowledge base on CORDs that translates existing disease guidelines into personalized patient recommendations and can be used as part of mHealth decision support systems. Multiple aspects needing improvement have already been identified in use case testing and an initial internal and external assessment process, reinforcing the relevance of conducting such evaluations before clinical use. Future studies should further assess the adequacy of the personalized recommendations in real life using longitudinal data and patient feedback. The development of this sound, evidence-based knowledge base to be included in a computerized decision support system was a long and iterative process but has been shown to be feasible if a multidisciplinary and motivated team is available. This process could be replicated in other clinical areas.

## Appendix

Multimedia Appendix 1 Harmonized level of evidence and corresponding classification systems for each of the source recommendations. Only those that used at least 1 classification system (evidence level or grade of the recommendation) were included in the table.

Multimedia Appendix 2 Description of the identified recommendations according to the source reference considering all those that were identified in the initial selection and those that were selected for further implementation.

Multimedia Appendix 3 Summary description of the logical rules and personalized recommendations (n=405).

Multimedia Appendix 4 Description of the use case and number of recommendations generated by (1) the variable irrespective of the answer, (2) the specific answer, and (3) the variable and answer within this specific use case.

Multimedia Appendix 5 Description of Control and Burden of Asthma and Rhinitis participants, including stratification by the number of triggered personalized recommendations.

## Abbreviations

AIRDOC: Aplicação móvel Inteligente para suporte individualizado e monitorização da função e sons Respiratórios de Doentes Obstrutivos Crónicos (mobile application for individualized support and monitoring of the respiratory function and sounds of chronic obstructive patients)
API: application programming interface
CDSS: clinical decision support system
COPD: chronic obstructive pulmonary disease
CORD: chronic obstructive respiratory disease
GINA: Global Initiative for Asthma
GOLD: Global Initiative for Chronic Obstructive Lung Disease
FHIR: Fast Healthcare Interoperability Resources
HLE: harmonized level of evidence
ICAR: Control and Burden of Asthma and Rhinitis
mHealth: mobile health

## Data Availability

The knowledge base generated and analyzed during this study is available from the corresponding author upon reasonable request.
