# Supplementary material for: Multidisciplinary Development and Initial Validation of a Clinical Knowledge Base on Chronic Respiratory Diseases for mHealth Decision Support Systems
Source: J Med Internet Res. 2023 Dec 13;25:e45364. doi: 10.2196/45364 (PMC10753423; doi:10.2196/45364)
Supplement: Multimedia Appendix 1 [file jmir_v25i1e45364_app1.docx]

Supplementary Table 1: Harmonized level of evidence (HLE) and corresponding classification systems in each of the source recommendations. Only those that used at least one classification system (evidence level or grade of the recommendation) were included in the table.

| **DEFINITION** | **HLE** | **GINA 2018**[1] | **GOLD 2018**[2] | **COPDX 2018**[3] | **BTS (2009, 2013, 2015, 2016)** [4-7] | **NOC (2013 and above)** [8-10] | **KNGF 2008**[11] | **ARIA 2010**[12] | **ACP 2013**[13] | **ACR 2010**[14] | **ACSM (2009, 2011)** [15, 16] | **ACCP/CTS 2015**[17] |
| --- | --- | --- | --- | --- | --- | --- | --- | --- | --- | --- | --- | --- |
| Evidence is from endpoints of well-designed RCTs or meta-analyses of relevant studies that provide a consistent pattern of findings in the population for which the recommendation is made. Category A requires substantial numbers of studies involving substantial numbers of participants. | **A** | A | A | I | 1++; 1+ (A) | A | A1; A2 | High | High | A | A | High (A) |
| Evidence is from endpoints of intervention studies that include only a limited number of patients, post hoc or subgroup analysis of RCTs or meta-analysis of such RCTs. In general, Category B pertains when few randomized trials exist, they are small in size, they were under-taken in a population that differs from the target population of the recommendation, or the results are somewhat inconsistent. | **B** | B | B | II | 1-; 2++ (B) | B | B | Moderate | Moderate | B | B | Moderate (B) |
| Evidence is from outcomes of uncontrolled or non-randomized trials or from observational studies. | **C** | C | C | III-1; III-2; III-3; IV | 2+; 2 (C) | C | C | Low | Low | B | C | Low (C) |
| This category is used only in cases where the provision of some guidance was deemed valuable but the clinical literature addressing the subject was insufficient to justify placement in one of the other categories. The Panel Consensus is based on clinical experience or knowledge that does not meet the above listed criteria. | **D** | D | D |  | 3; 4 (D) | C | D | Very low | Insufficient | C | D | Insufficient |
| ACCP: American College of Chest Physicians; ACP: American College of Physicians; ACR: American College of Rheumatology; ACSM: American College of Sports Medicine; ACPRC: Association of Chartered Physiotherapists in Respiratory Care; ARIA: Allergic Rhinitis and its Impact on Asthma; BTS: British Thoracic Society; COPDX: Australian and New Zealand Guidelines for the management of Chronic Obstructive Pulmonary Disease; CTS: Canadian Thoracic Society; GINA: Global Initiative for Asthma; GOLD: Global Initiative for Chronic Obstructive Lung Disease; KNGF: The Royal Dutch Society for Physical Therapy; NAEPP: National Asthma Education and Prevention Program; NOC: Clinical Practice Guideline, *Norma de Orientação Clínica* | | | | | | | | | | | | |

## References

1. Global Initiative for Asthma. Global Strategy for Asthma Management and Prevention. 2018; Available from: [www.ginasthma.org](https://d.docs.live.net/d25c1586a34ad810/Documentos/Projetos%20em%20curso/AIRDOC/Artigo%20Matriz_JMIR/JMIR_Submissão%201/www.ginasthma.org).

2. Global Initiative for Chronic Obstructive Lung D. Global Strategy for the Diagnosis, Management and Prevention of COPD, Global Initiative for Chronic Obstructive Lung Disease (2018 Report). 2018.

3. Yang I, Dabscheck E, Brown J, Jenkins S, McDonald C, McDonald V, et al. The COPD-X Plan: Australian and New Zealand Guidelines for the management of Chronic Obstructive Pulmonary Disease 2018. Version 2.53, March 2018. Brisbane: Lung Foundation Australia; 2018; Available from: <https://copdx.org.au/>.

4. British Thoracic Society, Scottish Intercollegiate Guidelines Network. SIGN 153 - British guideline on the management of asthma: A national clinical guideline. 2016; Available from: [www.brit-thoracic.org.uk](https://d.docs.live.net/d25c1586a34ad810/Documentos/Projetos%20em%20curso/AIRDOC/Artigo%20Matriz_JMIR/JMIR_Submissão%201/www.brit-thoracic.org.uk)

5. Bott J, Blumenthal S, Buxton M, Ellum S, Falconer C, Garrod R, et al. Guidelines for the physiotherapy management of the adult, medical, spontaneously breathing patient. Thorax. 2009 May;64 Suppl 1:i1-51. PMID: 19406863. doi: 10.1136/thx.2008.110726.

6. Hardinge M, Annandale J, Bourne S, Cooper B, Evans A, Freeman D, et al. British Thoracic Society guidelines for home oxygen use in adults: accredited by NICE. Thorax. 2015;70(Suppl 1):i1-i43. doi: 10.1136/thoraxjnl-2015-206865.

7. Bolton CE, Bevan-Smith EF, Blakey JD, Crowe P, Elkin SL, Garrod R, et al. British Thoracic Society guideline on pulmonary rehabilitation in adults. Thorax. 2013 Sep;68 Suppl 2:ii1-30. PMID: 23880483. doi: 10.1136/thoraxjnl-2013-203808.

8. Direção-Geral da Saúde. Monitorização e Tratamento Para o Controlo da Asma na Criança, no Adolescente e no Adulto (NOC 006/2018 de 26/02/2018). 2018; Available from: <https://normas.dgs.min-saude.pt/wp-content/uploads/2019/09/Monitorizacao-e-Tratamento-Para-o-Controlo-da-Asma-na-Crianca-no-Adolescente-e-no-Adulto.pdf>.

9. Direção-Geral da Saúde. Diagnóstico e Tratamento da Doença Pulmonar Obstrutiva Crónica (NOC 028/2011 atualizada a 10/09/2013). 2013; Available from: <https://nocs.pt/diagnostico-e-tratamento-dpoc/>.

10. Direção-Geral da Saúde. Cuidados Respiratórios Domiciliários: Prescrição de Ventiloterapia e outros Equipamentos (NOC 022/2011 atualizada a 11/09/2015). 2015; Available from: <https://normas.dgs.min-saude.pt/2011/09/28/cuidados-respiratorios-domiciliarios-prescricao-de-ventiloterapia-e-outros-equipamentos/>.

11. Gosselink R, Langer D, Burtin C, Probst V, Hendriks E, van der Schans C. Clinical practice guideline for physical therapy in patients with COPD - practice guidelines. Suppl Dutch J Phys Ther. 2008 01/01.

12. Brozek JL, Bousquet J, Baena-Cagnani CE, Bonini S, Canonica GW, Casale TB, et al. Allergic Rhinitis and its Impact on Asthma (ARIA) guidelines: 2010 revision. J Allergy Clin Immunol. 2010 Sep;126(3):466-76. PMID: 20816182. doi: 10.1016/j.jaci.2010.06.047.

13. Qaseem A, Holty JE, Owens DK, Dallas P, Starkey M, Shekelle P. Management of obstructive sleep apnea in adults: A clinical practice guideline from the American College of Physicians. Ann Intern Med. 2013 Oct 1;159(7):471-83. PMID: 24061345. doi: 10.7326/0003-4819-159-7-201310010-00704.

14. Grossman JM, Gordon R, Ranganath VK, Deal C, Caplan L, Chen W, et al. American College of Rheumatology 2010 recommendations for the prevention and treatment of glucocorticoid-induced osteoporosis. Arthritis Care Res (Hoboken). 2010 Nov;62(11):1515-26. PMID: 20662044. doi: 10.1002/acr.20295.

15. Chodzko-Zajko WJ, Proctor DN, Fiatarone Singh MA, Minson CT, Nigg CR, Salem GJ, et al. American College of Sports Medicine position stand. Exercise and physical activity for older adults. Med Sci Sports Exerc. 2009 Jul;41(7):1510-30. PMID: 19516148. doi: 10.1249/MSS.0b013e3181a0c95c.

16. Garber CE, Blissmer B, Deschenes MR, Franklin BA, Lamonte MJ, Lee IM, et al. American College of Sports Medicine position stand. Quantity and quality of exercise for developing and maintaining cardiorespiratory, musculoskeletal, and neuromotor fitness in apparently healthy adults: guidance for prescribing exercise. Med Sci Sports Exerc. 2011 Jul;43(7):1334-59. PMID: 21694556. doi: 10.1249/MSS.0b013e318213fefb.

17. Criner GJ, Bourbeau J, Diekemper RL, Ouellette DR, Goodridge D, Hernandez P, et al. Prevention of acute exacerbations of COPD: American College of Chest Physicians and Canadian Thoracic Society Guideline. Chest. 2015 Apr;147(4):894-942. PMID: 25321320. doi: 10.1378/chest.14-1676.
