# Supplementary material for: Multidisciplinary Development and Initial Validation of a Clinical Knowledge Base on Chronic Respiratory Diseases for mHealth Decision Support Systems
Source: J Med Internet Res. 2023 Dec 13;25:e45364. doi: 10.2196/45364 (PMC10753423; doi:10.2196/45364)
Supplement: Multimedia Appendix 2 [file jmir_v25i1e45364_app2.docx]

Supplementary Table 2: Description of the identified recommendations according to the source reference, considering all that were identified in the initial selection and those that were selected for further implementation.

|  |  | **All / Initial selection**  **(n=667)** | | **Selected for implementation**  **(n=358)** | | | |  |  |
| --- | --- | --- | --- | --- | --- | --- | --- | --- | --- |
|  |  | **n** | **(%)** | **n** | **(%)** | | **(% of the initial selection)** | |  |
| **Source document reference (stratified by clinical area)** | | | | | | | | | |
| Asthma | | 302 | (45.3) | 164 | | (45.8) | (54.3) | |  |
|  | *GINA 2018 (including appendix)* | 188 | (28.2) | 111 | (31.0) | | (59.0) | |  |
|  | *BTS 2016* | 69 | (10.3) | 31 | (8.7) | | (44.9) | |  |
|  | *NICE 2017* | 26 | (3.9) | 10 | (2.8) | | (38.5) | |  |
|  | *PNDR 2013* | 12 | (1.8) | 7 | (2.0) | | (58.3) | |  |
|  | *NOC 2018* | 6 | (0.9) | 4 | (1.1) | | (66.7) | |  |
|  | *Walters 2005* | 1 | (0.1) | 1 | (0.3) | | (100.0) | |  |
| Asthma and pregnancy | | 1 | (0.1) | 1 | | (0.3) | (100.0) | |  |
|  | *NAEPP 2004* | 1 | (0.1) | 1 | (0.3) | | (100.0) | |  |
| COPD | | 250 | (37.5) | 135 | | (37.7) | (54.0) | |  |
|  | *GOLD 2018* | 107 | (16.0) | 51 | (14.2) | | (47.7) | |  |
|  | *COPDX 2018* | 75 | (11.2) | 47 | (13.1) | | (62.7) | |  |
|  | *NICE 2018* | 45 | (6.7) | 22 | (6.1) | | (48.9) | |  |
|  | *NOC 2013* | 23 | (3.4) | 15 | (4.2) | | (65.2) | |  |
| COPD exacerbation | | 1 | (0.1) | 1 | | (0.3) | (100.0) | |  |
|  | *ACCP/CTS 2015* | 1 | (0.1) | 1 | (0.3) | | (100.0) | |  |
| Comorbidity - allergic rhinitis | | 29 | (4.3) | 6 | | (1.7) | (20.7) | |  |
|  | *ARIA 2001* | 10 | (1.5) | 4 | (1.1) | | (40.0) | |  |
|  | *ARIA 2010* | 9 | (1.3) | 2 | (0.6) | | (22.2) | |  |
| Comorbidity - OSA | | 8 | (1.2) | 3 | | (0.8) | (37.5) | |  |
|  | *ACP 2013* | 5 | (0.7) | 3 | (0.8) | | (60.0) | |  |
|  | *DGS 2016* | 2 | (0.3) | 0 | (0.0) | | (0.0) | |  |
|  | *Kribbs 1993* | 1 | (0.1) | 0 | (0.0) | | (0.0) | |  |
| Comorbidity – respiratory infections | | 1 | (0.1) | 1 | | (0.3) | (100.0) | |  |
|  | *NOC 2011* | 1 | (0.1) | 1 | (0.3) | | (100.0) | |  |
| Breathing exercises | | 13 | (1.9) | 11 | | (3.1) | (84.6) | |  |
|  | *BTS/ACPRC 2009* | 7 | (1.0) | 7 | (2.0) | | (100.0) | |  |
|  | *BTS 2013* | 6 | (0.9) | 4 | (1.1) | | (66.7) | |  |
| Oxygen therapy and ventilatory support | | 19 | (2.8) | 0 | | (0.0) | (0.0) | |  |
|  | *BTS 2015* | 18 | (2.7) | 0 | (0.0) | | (0.0) | |  |
|  | *NOC 2015* | 1 | (0.1) | 0 | (0.0) | | (0.0) | |  |
| Adherence to treatment | | 14 | (2.1) | 2 | | (0.6) | (14.3) | |  |
|  | *NICE 2009* | 14 | (2.1) | 2 | (0.6) | | (14.3) | |  |
| Physical activity and exercise | | 35 | (5.2) | 32 | | (8.9) | (91.4) | |  |
|  | *ACSM 2011* | 17 | (2.5) | 17 | (4.7) | | (100.0) | |  |
|  | *KNGF 2008* | 7 | (1.0) | 6 | (1.7) | | (85.7) | |  |
|  | *PA 2018* | 6 | (0.9) | 6 | (1.7) | | (100.0) | |  |
|  | *ACSM 2009* | 5 | (0.7) | 3 | (0.8) | | (60.0) | |  |
| Comorbidity - osteoporosis | | 4 | (0.6) | 2 | | (0.6) | (50.0) | |  |
|  | *ACR 2010* | 4 | (0.6) | 2 | (0.6) | | (50.0) | |  |

ACCP: American College of Chest Physicians; ACP: American College of Physicians; ACR: American College of Rheumatology; ACSM: American College of Sports Medicine; ACPRC: Association of Chartered Physiotherapists in Respiratory Care; ARIA: Allergic Rhinitis and its Impact on Asthma; BTS: British Thoracic Society; COPDX: Australian and New Zealand Guidelines for the management of Chronic Obstructive Pulmonary Disease; CTS: Canadian Thoracic Society; DGS: Directorate-General of Health, *Direção-Geral da Saúde*; GINA: Global Initiative for Asthma; GOLD: Global Initiative for Chronic Obstructive Lung Disease; KNGF: The Royal Dutch Society for Physical Therapy; NAEPP: National Asthma Education and Prevention Program; NICE: National Institute for Health and Care Excellence; NOC: Clinical Practice Guideline, *Norma de Orientação Clínica*; PNDR: National Programme for Respiratory Diseases, *Programa Nacional para as Doenças Respiratórias*; PA: Physical Activity; SIGN: Scottish Intercollegiate Guidelines Network
