# Supplementary material for: Multidisciplinary Development and Initial Validation of a Clinical Knowledge Base on Chronic Respiratory Diseases for mHealth Decision Support Systems
Source: J Med Internet Res. 2023 Dec 13;25:e45364. doi: 10.2196/45364 (PMC10753423; doi:10.2196/45364)
Supplement: Multimedia Appendix 3 [file jmir_v25i1e45364_app3.docx]

Supplementary Table 3: Summary description of the logical rules and personalized recommendations (n=405).

|  |  | **n** | **(%)** |
| --- | --- | --- | --- |
| **Number of variables used to build a logical rule** | |  |  |
|  | Mean (SD) number of variables | 3.5 | (1.8) |
|  | 1 variable | 34 | (8.4) |
|  | 2 variables | 105 | (25.9) |
|  | 3 variables | 108 | (26.7) |
|  | 4 variables | 54 | (13.3) |
|  | 5 variables | 41 | (10.1) |
|  | ≥6 variables | 63 | (15.6) |
| **Most frequently used variables (top 10)** | |  |  |
|  | Healthy (Yes/No) | 364 | (89.9) |
|  | Self-reported asthma (Yes/No) | 197 | (48.6) |
|  | Self-reported COPD (Yes/No) | 176 | (43.5) |
|  | Number of medications (general) | 49 | (12.1) |
|  | Number of medications for respiratory diseases | 47 | (11.6) |
|  | Age (in years) | 45 | (11.1) |
|  | Current pregnancy (Yes/No) | 43 | (10.6) |
|  | Number of inhalers | 30 | (7.4) |
|  | Presence of rhinitis (Yes/No) | 24 | (5.9) |
|  | Number of control medications for respiratory diseases | 18 | (4.4) |
| **Most frequently used logical conditions ^a^ (top 5)** | |  |  |
|  | Healthy = No AND Self-reported COPD = Yes | 62 | (15.3) |
|  | Healthy = No AND Self-reported Asthma = Yes | 36 | (8.9) |
|  | Age (in years) > 18 AND Age (in years) ≤ 65 | 16 | (4.0) |
|  | Current pregnancy = Yes | 9 | (2.2) |
|  | Healthy = No AND Self-reported Asthma = Yes AND Mite allergy = Yes | 7 | (1.7) |
| **Priority level assigned to the personalized recommendation** | |  |  |
|  | 1 (highest) | 47 | (11.6) |
|  | 2 | 102 | (25.2) |
|  | 3 | 116 | (28.6) |
|  | 4 | 79 | (19.5) |
|  | 5 (lowest) | 61 | (15.1) |

^a^ combinations of variables and logical operators (excluding the recommendation itself)
