# Supplementary material for: Multidisciplinary Development and Initial Validation of a Clinical Knowledge Base on Chronic Respiratory Diseases for mHealth Decision Support Systems
Source: J Med Internet Res. 2023 Dec 13;25:e45364. doi: 10.2196/45364 (PMC10753423; doi:10.2196/45364)
Supplement: Multimedia Appendix 4 [file jmir_v25i1e45364_app4.docx]

Supplementary Table 4: Description of the use case and number of recommendations generated by A) the variable, irrespective of the answer; B) the specific answer; and C) the variable and answer within this specific use case.

| **Variable** | **Use case characteristics** | | **A) Recommendations that use this variable (n=405)** | | **B)**  **Recommendations potentially generated with the specific answer (n=405)** | | **C)**  **Recommendations generated within the use case (n=78) ^a^** | |
| --- | --- | --- | --- | --- | --- | --- | --- | --- |
|  |  | | **n** | **(%)** | **n** | **(%)** | **n** | **(%)** |
| **Demographics** | |  |  |  |  |  |  |  |
| Sex | Male | | 17 | (4.2) | 1 | (0.2) | 0 | (0.0) |
| Age (in years) | 18 | | 45 | (11.1) | 9 | (2.2) | 5 | (6.4) |
| BMI (Kg/m^2^) | 21 (180cm; 70Kg) | | 17 | (4.2) | 1 | (0.2) | 0 | (0.0) |
| **Environment / Habits** |  | |  |  |  |  |  |  |
| Current smoking habits | Non-smoker | | 14 | (3.5) | 3 | (0.7) | 0 | (0.0) |
| Nr cigarettes/day | 0 | | 9 | (2.2) | 0 | (0.0) | 0 | (0.0) |
| Nr packs/year | 0 | | 2 | (0.5) | 0 | (0.0) | 0 | (0.0) |
| Exposure to tobacco smoke | No | | 14 | (3.5) | 3 | (0.7) | 0 | (0.0) |
| **Diagnosis/Comorbidities** |  | |  |  |  |  |  |  |
| Self-reported asthma | Yes | | 197 | (48.6) | 182 | (44.9) | 71 | (91.0) |
| Self-reported rhinosinusitis | Yes | | 6 | (1.5) | 6 | (1.5) | 1 | (1.3) |
| Self-reported rhinitis | Yes | | 24 | (5.9) | 7 | (1.7) | 5 | (6.4) |
| Rhinitis classification | Persistent | | 2 | (0.5) | 2 | (0.5) | 2 | (2.6) |
| Anxiety symptoms (HADS-A) | 9 | | 14 | (3.5) | 14 | (3.5) | 4 | (5.1) |
| Depression symptoms (HADS-D) | 9 | | 13 | (3.2) | 13 | (3.2) | 4 | (5.1) |
| **Medication in use** |  | |  |  |  |  |  |  |
| Number of medications |  | |  |  |  |  |  |  |
| Total medications | 3 | | 49 | (12.1) | 49 | (12.1) | 12 | (15.4) |
| Medication for respiratory disease | 2 | | 47 | (11.6) | 46 | (11.4) | 9 | (11.5) |
| Control medication for respiratory disease | 1 | | 18 | (4.5) | 14 | (3.5) | 2 | (2.6) |
| Inhalers | 2 | | 30 | (7.4) | 30 | (7.4) | 6 | (7.7) |
| ICS | 1 | | 25 | (6.2) | 15 | (3.7) | 1 | (1.3) |
| Dose of ICS | Low | | 2 | (0.5) | 1 | (0.2) | 0 | (0.0) |
| SABA in the action plan | Yes | | 5 | (1.2) | 5 | (1.2) | 0 | (0.0) |
| SABA inhalations per day | >1 | | 1 | (0.2) | 1 | (0.2) | 0 | (0.0) |
| Nasal corticosteroids | Yes | | 0 | (0.0) | 0 | (0.0) | 0 | (0.0) |
| Adherence to medication | <50% | | 17 | (4.2) | 7 | (1.7) | 2 | (2.6) |
| Inhaler technique | No incorrections | | 12 | (3.0) | 3 | (0.7) | 0 | (0.0) |
| Number of inhaler technique assessments | 1 | | 7 | (1.7) | 5 | (1.2) | 0 | (0.0) |
| Weeks since last inhaler technique check-up | 20 | | 16 | (4.0) | 13 | (3.2) | 4 | (5.1) |
| **Symptoms control** |  | |  |  |  |  |  |  |
| Asthma control (CARAT score) | 13 (uncontrolled) | | 18 | (4.4) | 17 | (4.2) | 5 | (6.4) |
| Borg scale for dyspnoea | 4 | | 4 | (1.0) | 4 | (1.0) | 0 | (0.0) |
| Activity Limitations (CAT) | >3 | | 8 | (2.0) | 2 | (0.5) | 0 | (0.0) |
| Current asthma exacerbation | Yes | | 8 | (2.0) | 8 | (2.0) | 3 | (3.8) |
| Hospitalizations/ER care in past year | 0 | | 12 | (3.0) | 1 | (0.2) | 0 | (0.0) |
| **Objective measurements** |  | |  |  |  |  |  |  |
| Sensitization to at least one allergen | Yes | | 23 | (5.7) | 23 | (5.7) | 12 | (15.4) |
| Sensitization to at least one indoor allergen | Yes | | 20 | (4.9) | 20 | (4.9) | 10 | (12.8) |
| Pollen allergy | Yes | | 1 | (0.2) | 1 | (0.2) | 0 | (0.0) |
| Mite allergy | Yes | | 9 | (2.2) | 9 | (2.2) | 8 | (10.3) |
| FEV1 (% of predicted), pre-BD | 115% | | 18 | (4.4) | 8 | (2.0) | 0 | (0.0) |

^a^ some of the potentially generated recommendations (B) could not be triggered because other necessary variables had no available information or had an answer that made the recommendation unapplicable to this specific patient.

BMI - Body Mass Index; CARAT - Control of Allergic Rhinitis and Asthma Test; CAT – COPD Assessment Test; ER, Emergency Room; FEV1, Forced expiratory volume in 1 second; BD, bronchodilation test
