# Supplementary material for: Multidisciplinary Development and Initial Validation of a Clinical Knowledge Base on Chronic Respiratory Diseases for mHealth Decision Support Systems
Source: J Med Internet Res. 2023 Dec 13;25:e45364. doi: 10.2196/45364 (PMC10753423; doi:10.2196/45364)
Supplement: Multimedia Appendix 5 [file jmir_v25i1e45364_app5.docx]

Supplementary Table 5: Description of ICAR participants, including stratification by the number of triggered personalized recommendations.

| **Patient characteristic** | | **All ICAR participants** (n=728) | | **≤25 triggered recommendations ^a^** (n=551) | | **>25 triggered recommendations** (n=177) | | **p-value ^b^** |
| --- | --- | --- | --- | --- | --- | --- | --- | --- |
|  |  | **n** | **(%)** | **n** | **(%)** | **n** | **(%)** |  |
| **Sex**, male | | 267 | (36.7%) | 197 | (35.8%) | 70 | (39.5%) | 0.371 |
| **Age**, years, mean(SD) | | 43.9 | 15.1 | 44.1 | 15.1 | 43.1 | 15.3 | 0.423 |
| **BMI**, Kg/m2, mean(SD) | | 25.9 | 4.7 | 25.7 | 4.5 | 26.6 | 5.1 | **0.037** |
| **Healthy** | | 104 | (14.3%) | 104 | (18.9%) | 0 | (0.0%) | **<0.001** |
| **Respiratory diseases** | |  |  |  |  |  |  |  |
|  | COPD | 6 | (0.8%) | 0 | (0.0%) | 6 | (3.4%) | **<0.001** |
|  | Asthma | 169 | (23.2%) | 0 | (0.0%) | 169 | (95.5%) | **<0.001** |
|  | Rhinitis | 518 | (71.2%) | 358 | (65.0%) | 160 | (90.4%) | **<0.001** |
|  | Rhinosinusitis | 211 | (29.0%) | 144 | (26.1%) | 67 | (37.9%) | **0.003** |
|  | Other respiratory diseases | 69 | (9.5%) | 69 | (12.5%) | 0 | (0.0%) | **<0.001** |
| **Comorbidities** | |  |  |  |  |  |  |  |
|  | Obstructive sleep apnoea | 6 | (0.8%) | 2 | (0.4%) | 4 | (2.3%) | **0.033** |
|  | Conjunctivitis | 377 | (52.5%) | 264 | (48.6%) | 113 | (64.6%) | **<0.001** |
|  | Food allergy | 16 | (2.2%) | 9 | (1.7%) | 7 | (4.1%) | 0.073 |
|  | Gastroesophageal reflux disease | 115 | (16.0%) | 78 | (14.3%) | 37 | (21.1%) | **0.031** |
|  | Major psychological problems | 131 | (18.1%) | 106 | (19.3%) | 25 | (14.4%) | 0.144 |
|  | Cardiovascular disease | 132 | (18.3%) | 100 | (18.3%) | 32 | (18.3%) | 1.000 |
|  | Diabetes mellitus | 32 | (7.7%) | 28 | (9.0%) | 4 | (3.9%) | 0.134 |
| **Pregnant** | | 4 | (1.2%) | 1 | (0.4%) | 3 | (3.8%) | **0.042** |
| **Breastfeeding** | | 6 | (1.3%) | 4 | (1.1%) | 2 | (1.9%) | 0.627 |
| **Currently smoking** | | 160 | (22.1%) | 131 | (23.9%) | 29 | (16.6%) | **0.047** |
| **Number of packs/year**, mean(SD) | | 15.5 | 17.3 | 15.6 | 16.1 | 15.2 | 21 | 0.876 |
| **Exposed to secondhand tobacco smoke** | | 330 | (57.6%) | 246 | (57.2%) | 84 | (58.7%) | 0.770 |
| **Sensitized to at least one allergen** | | 432 | (59.7%) | 290 | (52.9%) | 142 | (80.7%) | **<0.001** |
| **Respiratory infection in the past 3 months** | | 206 | (28.5%) | 151 | (27.5%) | 55 | (31.4%) | 0.336 |
| **Respiratory symptoms** | |  |  |  |  |  |  |  |
|  | Dry cough | 105 | (14.9%) | 60 | (11.1%) | 45 | (27.6%) | **<0.001** |
|  | Dyspnoea | 193 | (26.5%) | 71 | (12.9%) | 122 | (68.9%) | **<0.001** |
|  | Sputum | 185 | (25.5%) | 100 | (18.2%) | 85 | (48.3%) | **<0.001** |
|  | CARAT score, mean (SD) | 21.6 | 5.2 | 22.5 | 4.7 | 20.2 | 5.5 | **<0.001** |
|  | Uncontrolled respiratory symptoms (CARAT≤24) | 297 | (70.9%) | 167 | (64.5%) | 130 | (81.3%) | **<0.001** |
| **FEV1 % predicted**, mean (SD) | | 97.4 | 15.5 | 100.2 | 13.8 | 88.6 | 17.3 | **<0.001** |
| **Medication for respiratory diseases** | |  |  |  |  |  |  |  |
|  | At least one medication for respiratory disease | 333 | (45.7%) | 193 | (35.0%) | 140 | (79.1%) | **<0.001** |
|  | At least one control medication for respiratory disease | 92 | (12.6%) | 8 | (1.5%) | 84 | (47.5%) | **<0.001** |
|  | At least one inhaler | 119 | (16.3%) | 9 | (1.6%) | 110 | (62.1%) | **<0.001** |
|  | At least one inhaled corticosteroid | 82 | (11.3%) | 6 | (1.1%) | 76 | (42.9%) | **<0.001** |
|  | At least one prescribed rescue inhaler | 57 | (7.9%) | 7 | (1.3%) | 50 | (28.6%) | **<0.001** |
|  | Current oral corticosteroid intake | 6 | (0.8%) | 1 | (0.2%) | 5 | (2.9%) | **0.001** |

^a^ one individual had no triggered recommendations, an 18-years old female diagnosed with conjunctivitis (without rhinitis, asthma, or other respiratory diseases) and who was a current smoker with a low lifetime tobacco exposure (0.5 packs/year).

^b^ using Fisher’s exact test and independent sample t-test for categorical and continuous variables, respectively.
